# Contextual and Auditory Fear Conditioning Continue to Emerge during the Periweaning Period in Rats

**DOI:** 10.1371/journal.pone.0100807

**Published:** 2014-06-30

**Authors:** Michael A. Burman, Kristen J. Erickson, Alex L. Deal, Rose E. Jacobson

**Affiliations:** 1 Department of Psychology, Center for Excellence in the Neurosciences, University of New England, Biddeford, Maine, United States of America; 2 Department of Biology, University of New England, Biddeford, Maine, United States of America; University of Lethbridge, Canada

## Abstract

Anxiety disorders often emerge during childhood. Rodent models using classical fear conditioning have shown that different types of fear depend upon different neural structures and may emerge at different stages of development. For example, some work has suggested that contextual fear conditioning generally emerges later in development (postnatal day 23–24) than explicitly cued fear conditioning (postnatal day 15–17) in rats. This has been attributed to an inability of younger subjects to form a representation of the context due to an immature hippocampus. However, evidence that contextual fear can be observed in postnatal day 17 subjects and that cued fear conditioning continues to emerge past this age raises questions about the nature of this deficit. The current studies examine this question using both the context pre-exposure facilitation effect for immediate single-shock contextual fear conditioning and traditional cued fear conditioning using Sprague-Dawley rats. The data suggest that both cued and contextual fear conditioning are continuing to develop between PD 17 and 24, consistent with development occurring the in essential fear conditioning circuit.

## Introduction

The time course of limbic-system development and the ontogeny of fear and anxiety are critical for our understanding of anxiety disorders. Indeed, there is now growing appreciation that anxiety may be a developmental disorder, or at least a disorder with a large developmental component [1]–[3]. The average age of onset for anxiety disorders in humans is 12 years old, with some forms, such as specific phobias, emerging closer to age 6 [4], [5]. It is probably not a coincidence that fear conditioning appears to mature in humans just prior to this, between the ages of 2 and 6 [6]. Given the progress made in recent decades using rodent models of fear conditioning to elucidate the neural circuitry supporting fear in adult organisms, one might expect that this approach would also pay dividends in understanding the developmental emergence of these systems. Instead, the literature on the emergence of fear conditioning in rodents appears somewhat inconsistent.

Several studies suggest that the ability of rats to exhibit conditioned freezing to an explicit cue (such as a tone) emerges over a different time course during development than the ability to exhibit freezing to a context, such as the conditioning apparatus [7]–[10]. These types of learning have been shown to rely, in part, upon different neural substrates [11]–[13]. These studies suggest that explicit cue conditioning first emerges a couple of days after the relevant sensory systems come online [14]. Thus, conditioning to an auditory cue emerges around postnatal day (PD) 15 in rats, whereas conditioning to a visual cue emerges around PD 17. This type of learning can be shown, in adult subjects, to rely upon a fairly circumscribed set of neural structures primarily consisting of the amygdala as well as afferent and efferent pathways [15]–[17]. In contrast, the ability to exhibit enhanced freezing to a context is relatively impaired until PD 23–24 [7], [10], [14]. In addition, freezing to a context requires a set of neural structures including the dorsal hippocampus and associated regions of cortex [18]. These findings, in addition to biological evidence (for example, [19], [20]), have led some researchers to the conclusion that hippocampus development is the limiting factor in the relatively late emergence of contextual fear conditioning.

Other research suggests that contextual freezing may emerge earlier and can be observed under a variety of circumstances closer to PD 17 [9], [21]–[25]. An additional body of work, in tasks that allow the separation of the contextual learning from the fearful learning, suggests that contextual learning can occur around PD 17, but cannot be utilized for subsequent fear conditioning, latent inhibition or renewal until after weaning [26]–[28], although this may depend upon the specific nature of the context and task [29], [30]. Similar findings have now also been reported in spatial navigation tasks [31]. Thus, there appears be to some evidence that contextual learning may emerge prior to PD 23, although the implications of this for hippocampus function are unclear as these tasks may be solvable using a variety of cognitive and behavioral strategies.

In addition to evidence that contextual conditioning may emerge prior to weaning, there is also evidence that auditory conditioning may be continuing to develop later than previously believed. For example, when examining extinction of fear conditioning, Rick Richardson and colleagues report giving twice as many CS-US pairings to PD 16 subjects than to PD 23 subjects in order to achieve equivalent initial freezing, suggesting that PD 23 subjects otherwise demonstrate greater conditioning [32], [33]. Although they did not directly compare across ages, another study found that there is an almost 3-fold increase in freezing between PD 17 and 28 to a CS-context compound [25]. No such increase was found to the context alone, suggesting that CS-elicited freezing accounted for this effect. Overall then, the belief that auditory fear conditioning emerges prior to contextual fear conditioning appears to be challenged in two ways: contextual conditioning may be observed earlier and auditory fear conditioning may continue to strengthen later than previously believed.

To address this, our experiments will examine the emergence of contextual and auditory fear conditioning in the periweaning period using two behavioral approaches. Our first set of studies will examine the emergence of contextual conditioning using a contextual pre-exposure task. This capitalizes upon a pair of observations [34]–[36] that presenting a footshock immediately upon presentation to a context produces relatively weak (although still present, see [23], [37]) freezing compared to delaying the footshock by several seconds or minutes (termed the immediate-shock deficit) and that this deficit can be reversed by prior experience with the context (termed contextual pre-exposure facilitation). Thus, the contextual pre-exposure procedure allows the separation of the contextual and aversive learning across days and appears to be particularly sensitive to the effects of hippocampus manipulations because it precludes use of an elemental strategy [36]. Although there is now a body of work examining this task in developing rats [26], [36], [38]–[40], the current experiments were designed to replicate and expand upon the previous literature by examining additional behaviors, such as the latency to initiate freezing behavior and the average movement amplitude prior to the initial freezing bout in addition to percent freezing. In addition, the current experiments examined the effects of context exposure on single-shock context conditioning with a variety of placement-to-shock intervals to assess the robustness of the effect.

Our second set of experiments will assess the specificity of the deficit in pre-weaning subjects by examining the effects of age on a cued conditioning paradigm. A deficit in conditioning to the auditory cue in younger subjects would suggest that development is occurring in the basic fear conditioning circuit and may not be limited to the contextual learning system. Furthermore, during cued conditioning fear develops not only to the explicit cue, but also to the background context. Indeed, some studies have suggested that background conditioning is stronger than explicit contextual conditioning in pre-weaning subjects [22], [25]. Thus, these experiments will clarify the nature of the conditioning deficit in pre-weaning subjects, compared to their older counterparts. Together, these experiments will lead us to the conclusion that both auditory and contextual fear conditioning is relatively impaired in PD 17, compared to PD 24, rats.

## Experiment 1

These studies capitalized upon the pre-exposure reversal of the immediate shock deficit in contextual conditioning to examine the ontogeny of contextual fear conditioning.

### Materials and Methods

#### Ethics Statement

These studies were carried out in strict accordance with the recommendations in the Guide for the Care and Use of Laboratory Animals of the National Institutes of Health. The protocol was approved by the Institutional Animal Care and Use Committee of the University of New England (Approval Number: 2011-0705BUR). All efforts were made to minimize suffering.

#### Subjects

3 studies were conducted using rats at different ages. 100, 96 or 163 offspring from 11, 11, or 18 Timed-Pregnant Sprague-Dawley (Charles River) rats were used for Experiments 1–1, 1–2 and 1–3 respectively. Timed-Pregnant Sprague-Dawley rats arrived on either gestational day (GD) 12 or 18 and were checked daily for birth. All subjects were born on GD 21 or 22 and GD 22 was considered the day of birth (postnatal day 0) for all litters. Litters were housed in 43 cm×44 cm×20 cm transparent PET cages (Innovive, San Diego CA) in the University of New England rat vivarium. On postnatal day (PD) 3, litters were culled to 10 pups per litter (5 male and 5 female) whenever possible. All subjects lived with littermates and mother prior to weaning, which occurred on PD 21. Post-weaning subjects were housed with their same sex littermates (approximately 5 per cage). All subjects were maintained on a 12∶12 hour light/dark cycle and had free access to food and water at all times in the vivarium. At the end of the experiment, animals were euthanized by CO2 overdose.

#### Apparatus

Four Startfear chambers (Harvard Apparatus/Panlab model #58722) were used to collect all behavioral data [41]. The outer chamber consisted of a 67 cm×53 cm×55 cm insulated chamber with a dim light on the inside. The inner chambers consisted of a grid floor capable of delivering a foot shock and a speaker that played 65-dB white noise as background during all testing. The chambers were set up in two different contextual configurations as necessitated by separate ongoing experiments. Context A was a 25 cm×25 cm×25 cm box with three black walls, on transparent wall that was used as the chamber opening, a white ceiling with speaker, and a grid floor. It was cleaned using 70% ethanol between each run. Context B consisted of three white walls inside of which a transparent cylinder with 24 cm diameter sat, one transparent wall that was used as the chamber opening, a white ceiling with speaker, and a grid floor. Context B was cleaned with 1% ammonia between each run. The grid floor sat on load cells that detect movement through changes in relative weight positioning across the floor. Subjects could move freely within the apparatus while Startfear “Freezing” computer software recorded the relative weight displacement of the animal. Freezing behavior was defined as an absence of cage displacement above a threshold for a minimum of 500 ms, with the gain set to 4 and the low threshold also set to 4. The threshold was determined by pilot studies correlating activity measured by the computer with that of a trained observer (r = .85). The gain on the hardware was set to 8000. Activity was sampled every 50 ms.

#### Behavioral Testing

In order to compare to our previous work [26], [40], a similar three-day paradigm was used during each experiment with the goal of separating the formation of a contextual memory from the formation of a fearful memory. On the first day (pre-exposure), all subjects were transported into the lab in their home cages and stored in a holding room. Subjects were then individually removed from their home cage, weighed, and tail marked. They were then placed into a transparent 24 cm×18 cm×13 cm transport cage that had been washed out with either 70% ethanol (Context A) or 1% ammonia (Context B). The subjects were then transported into the conditioning room in groups of two to four subjects. Half of the subjects were then placed into a context for five minutes and their freezing was recorded (pre-exposure group) while the other subjects remained in their transport cages (no pre-exposure group). Subjects were then placed back into their transport cages, back into their home cage, and back to the vivarium for 24 hours.

On the second day (training), subjects were again transported into the lab in their home cages and stored in a holding room. Subjects were then individually removed from their home cage, weighed, and placed in a transport cage. They were then transported into the conditioning room in the same groups as the day before. All subjects were individually placed into the same context as the previous day where they received a 1.5 mA foot shock that was either immediate or had a 5, 10, 20, or 30 second delay (to compare with critical time points found by Fanselow, 1990 and our previous work) between the time that they were placed in the context and the time that they received the shock. This relatively high shock level is required to observe the pre-exposure facilitation effect when using freezing as the dependent measure [23]. All subjects were run one at a time to ensure that they received the shock after the correct delay time. Afterwards, subjects were removed from the context as quickly as possible and placed back into their transport cage. They were then transported back to the holding room, into their home cage, and into the vivarium for 24 hours.

On the third day (testing), subjects were again transported into the lab in their home cages and stored in a holding room. Subjects were individually removed from their home cage, weighed, and placed into a transport cage. They were then transported into the conditioning room in the same groups as the previous two days. All subjects were then placed into the same context as the previous day for five minutes during which their activity was digitized and recorded. Latency to first freezing bout, average movement amplitude during the initial movement bout, and percent freezing were recorded. Following this session, subjects were removed from the conditioning apparatus, placed back into their transport cage, and then back into their home cage.

Each experiment was run over 3–5 separate replications, the data of which was pooled to create the final dataset. The various behavioral groups were conducted in pseudo-random order (human designed and precluding repetition) and under the condition that each condition was run equally often in any given cage. For Experiment 1–1: 17/18/19, pre-exposure occurred on PD 17, conditioning on PD 18 and testing on PD 19. For Experiment 1–2: 24/25/26, pre-exposure occurred on PD 24, conditioning on PD 25 and testing on PD 26. For Experiment 1–3: 17/25/26, pre-exposure occurred on PD 17, conditioning on PD 25 and testing on PD 26.

#### Data Analysis and Statistics

The data was collected using Startfear “Freezing” computer software. The subjects in Experiment 1 were either pre-exposed, or not, to the context on Day 1 and given a single shock on Day 2 following various placement-to-shock delays. Subjects were randomly assigned to groups. In general, no more than 1 same-sex littermate was assigned to an experimental condition. In cases where this rule was accidentally deviated from, data from the subjects were averaged and treated statistically as a single observation. Subjects in each experiment were distributed across the groups as shown in Table 1.

**Table 1:** Experimental groups and design for Experiment 1.

Experiment 1–1: 17/18/19		
Placement-to-Shock Interval	Pre-exposure	Number of subjects
0	Yes	10(6 m, 4f)
0	No	8 (3 m, 5f)
5	Yes	9 (4 m, 5f)
5	No	11 (7 m, 4f)
10	Yes	9 (4 m, 5f)
10	No	9 (5 m, 4f)
20	Yes	8 (3 m, 5f)
20	No	11 (5 m, 6f)
30	Yes	9 (4 m, 5f)
30	No	9 (5 m, 4f)

Experiment 1–2: 24/25/26		
0	Yes	10 (6 m, 4f)
0	No	10 (4 m, 6f)
5	Yes	9 (5 m, 4f)
5	No	10 (5 m, 5f)
10	Yes	9 (6 m, 3f)
10	No	9 (4 m, 5f)
20	Yes	9 (4 m, 5f)
20	No	8 (4 m, 4f)
30	Yes	10 (4 m, 6f)
30	No	9 (4 m, 5f)

Experiment 1–3: 17/25/26		
0	Yes	14 (8 m, 6f)
0	No	14 (5 m, 8f)
5	Yes	16 (7 m, 9f)
5	No	17 (8 m, 9f)
10	Yes	16 (6 m, 10f)
10	No	13 (6 m, 7f)
20	Yes	14 (5 m, 9f)
20	No	15 (7 m, 8f)
30	Yes	12 (6 m, 6f)
30	No	15 (9 m, 6f)

We expected that our prepubescent subjects would not show any differences as a function of sex and that the different contexts would not affect conditioning. Indeed, initial omnibus ANOVAs showed no main effects or interactions with sex or cage (although it should be noted that these analyses are likely underpowered as we expected to collapse across these variables), therefore two-way ANOVAs (pre-exposed or not) X placement-to-shock interval (0, 5, 10, 20, or 30 s) were conducted using JMP10 for Macintosh to examine the effect of pre-exposure and placement-to-shock interval on initial movement, latency to freeze and percent freezing during the day of testing.

### Results and Discussion

**Figure 1:**
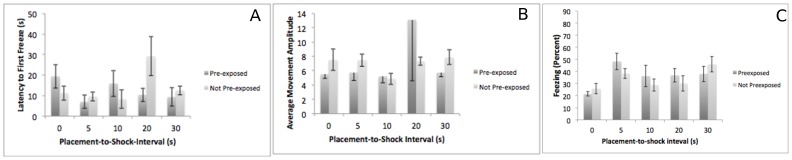
The results of Experiment 1–1 – contextual fear conditioning at PD 17/18/19. Subjects pre-exposed (dark bars) or not pre-exposed (light bars) to the conditioning context on day 1 do not differ in their latency to freeze (Panel A), their initial movement amplitude (Panel B) or in their freezing (Panel C) on test day.

Experiment 1–1: 17/18/19. Subjects were pre-exposed to a context or not on PD 17, given a shock on PD 18, and tested on PD 19. We expected that pre-exposure would fail to enhance conditioning in subjects conditioned at this age. The results supported this hypothesis (Figure 1). A 2 (pre-exposure) X 5 (placement-to-shock interval) ANOVA on latency to freeze demonstrated no main effect of pre-exposure and no main effect of placement-to-shock interval. The interaction approached significance F(4,84)  = 2.2 p = .07, however a Tukey's post-hoc test found no significant pairwise comparisons. Similarly, a 2 (pre-exposure) X 5 (placement-to-shock interval) ANOVA on movement amplitude during the initial movement bout found no main effect of pre-exposure, no main effect of placement-to-shock interval and no interaction. Finally, a 2 (pre-exposure) X 5 (placement-to-shock interval) ANOVA on percent freezing found a significant main effect of placement-to-shock interval F(4,84)  = 3.63, p<.05. A Tukey's post-hoc test found that the 0-s placement-to-shock interval differs significantly from the 5-s and 30-s intervals. No other differences were significant.

**Figure 2:**
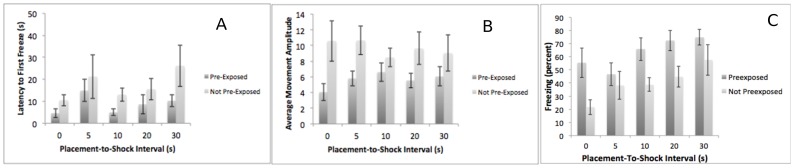
The results of Experiment 1–2 – contextual fear conditioning at PD 24/25/26. Subjects pre-exposed (dark bars) or not pre-exposed (light bars) to the conditioning context on day 1 significantly differ in their latency to freeze (Panel A), their initial movement amplitude (Panel B) and their freezing (Panel C) on test day.

Experiment 1–2: 24/25/26. Subjects were pre-exposed to a context or not on PD 24, given a shock on PD 25, and tested on PD 26. We expected that pre-exposure would enhance contextual conditioning at this age, but only at intermediate placement-to-shock intervals (Fanselow 1990). Instead, we saw a general facilitation of conditioning due to pre-exposure regardless of placement-to-shock interval (Figure 2). A 2 (pre-exposure) X 5 (placement-to-shock interval) ANOVA on latency to freeze demonstrated a significant main effect of pre-exposure F (1,82)  = 4.57, p<.05, with the pre-exposed subjects freezing faster. Neither the main effect of placement-to-shock interval nor the interaction was significant. Similarly, a 2 (pre-exposure) X 5 (placement-to-shock interval) ANOVA on initial movement amplitude demonstrated a significant main effect of pre-exposure F (1,82)  = 16.1, p<.01, with the pre-exposed subjects moving less. Neither the main effect of placement-to-shock interval nor the interaction was significant. Finally, a 2 (pre-exposure) X 5 (placement-to-shock interval) ANOVA on percent freezing found a main effect of placement-to-shock interval time F(4,83)  = 3.53, p<.05 and pre-exposure F(1,83)  = 17.35, p<.01, consistent with longer exposure to the context leading to greater freezing. The interaction was not significant. A Tukey's post-hoc test found that the 0-s and 30-s placement-to-shock intervals significantly different. No other differences were significant.

**Figure 3:**
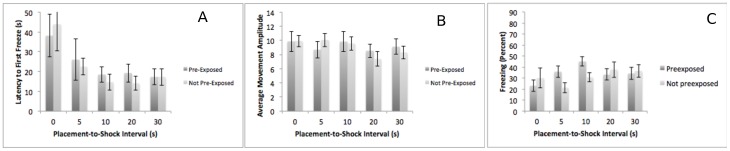
The results of Experiment 1–3 – contextual fear conditioning at PD 17/25/26. Subjects pre-exposed (dark bars) or not pre-exposed (light bars) to the conditioning context on day 1 do not differ in their latency to freeze (Panel A), initial movement amplitude (Panel B), or their freezing (Panel C) on test day. To specifically examine whether pre-exposure can have any effect on conditioning, as reported by Foster & Burman (2010), we separately examined the 5- and 10-s placement to shock intervals. Although only approaching significance at 5 s, there was a significant effect of pre-exposure at the 10-s placement-to-shock interval.

Experiment 1–3: 17/25/26. Subjects were pre-exposed to a context or not on PD 17, given a shock on PD 25, and tested on PD 26. Based on Foster & Burman (2010) we expected that subjects run in this paradigm would show enhanced fear conditioning based on pre-exposure, but only at moderate intervals. The results of Experiment 3 do not support this hypothesis (Figure 3). A 2 (pre-exposure) X 5 (placement-to-shock interval) ANOVA on latency to freeze demonstrated a significant main effect of placement-to-shock interval F (4,136)  = 4.09, p<.01. Neither the main effect of pre-exposure nor the interaction was significant. A Tukey's post-hoc test confirms that the 0-s placement to shock interval subjects took significantly longer to freeze than the 10-, 20-, and 30-s placement-to-shock interval subjects. No other pairwise comparisons were significant. A 2 (pre-exposure) X 5 (placement-to-shock interval) ANOVA on movement amplitude during the initial movement bout found no main effect of pre-exposure, no main effect of placement-to-shock interval and no interaction. Finally, a 2 (pre-exposure) X 5 (placement-to-shock interval) ANOVA found no significant main effects or interactions. The failure of pre-exposure to enhance contextual fear conditioning in these subjects appears inconsistent with the results presented in Foster & Burman (2010). To further explore this issue, we specifically examined the effects of pre-exposure at the 5- and 10-s placement to shock intervals using planned comparisons. Although only trending towards significance at the 5-s interval (p = .054), we did find a significant effect of pre-exposure at the 10-s interval (p<.05), suggesting that pre-exposure can facilitate freezing in this group under limited parametric circumstances.

## Experiment 2

Experiment 1 suggested that PD 17 subjects were impaired in contextual fear conditioning. In order to assess whether the observed differences in contextual conditioning due to age were specific to contextual learning or were a function of reduced fear conditioning overall, an additional experiment was conducted examining auditory fear conditioning and the concomitant background contextual conditioning.

### Materials and Methods

#### Subjects

Offspring (76 females and 71 males) from 18 litters of Timed-Pregnant Sprague-Dawley rats were used (conditioning with either a tone paired or unpaired 0.3 mA or 1.0 mA footshock). Husbandry conditions were exactly as above.

#### Apparatus

The conditioning apparatus was exactly as above.

#### Behavioral Testing

Rats were randomly assigned to one of two conditioning groups (paired or unpaired tone-shock) and each experiment had no more than one male and one female littermate per condition. Each cohort was run on 3 consecutive days and began on either PD 17 or PD 24. The same experimenter performed the three-day behavioral protocol between 8∶30 a.m. and 1∶00 p.m. each day with any given cohort. Start times for each day were within one hour of the start time of the first day. On each day, the home cage was brought into the lab. Rats were weighed, labeled (only on the first day), and placed into individual transport cages. The transport cages were 24 cm×18 cm×13 cm and made of clear plastic. All ambient sound was muted and the rats were transported to the testing room and placed into individual Startfear conditioning chambers. After each test, all rats were returned to their original cages. The experimental chambers were then cleaned thoroughly using Sparkleen and water, followed by either ethanol (Context A) or ammonia (Context B).

On the first day, (PD 17 or 24), animals in the “paired training” group were habituated to the experimental chamber for 5 minutes at the start of training. This habituation period was followed by 10 conditioning trials in which a 10-s 70-dB 4-Khz tone coterminated with an aversive stimulus (a 2-s 0.3 mA or 1.0-mA footshock). Trials were separated by randomly chosen intervals (mean  = 2.5 min, range  = 1.5–3.5 min). Animals in the “unpaired training” were also habituated to the experimental chamber for 5 minutes. However, the subsequent 10 auditory tones and 10 aversive stimuli were presented in random order. Any two stimuli were separated by a randomly chosen interval between 45 and 105 s (mean  = 75 s). Although our software continuously records movement, due to the confounding influence of footshock on freezing to the tone, we only report freezing for the 5 minutes of context exposure prior to the onset of the aversive stimulus.

The following day, rats underwent one of two testing conditions: “Context Testing: or “Novel Context and Tone Testing”. In “Context Testing,” the rats were returned to the same experimental chamber with the same contextual configuration for 5 minutes. Freezing behavior was recorded. In “Novel Context and Tone Testing,” rats were placed into a novel chamber, which was in the opposite contextual configuration, for 5 minutes during which freezing was recorded. This was followed by 10 exposures to the 10-s 4-Khz tone, separated by 20-s intervals. The software reports freezing behavior to the tone as percent freezing across all 10 trials. On the third day, rats were returned to the experiment and subjected to the alternate test. However these data are not included in the current report due to the difficulty in interpreting the data due to the potential effects of the first test session.

#### Data analysis and statistics

Rats were divided into 16 groups (see table 2). To reduce variability and account for differences in baseline freezing levels as a function of age, difference scores were created for both contextual (context test – habituation period freezing) and auditory (tone freezing – novel context freezing) indices of fear. Data were analyzed using JMP 10 software for macintosh. 2 (paired vs. unpaired) X 2 (shock level) X 2(age) ANOVAs were conducted on contextual and auditory difference scores respectively. Tukey's HSD post-hoc tests were conducted on subsets of the data following one- and two-way ANOVAs depending on the hypothesis being tested.

**Table 2:** Experimental groups and design for Experiment 2.

Group Name	Condition	Day 1 (PD 17 *or* 24)	Day 2 (PD 18 *or* 25)	Number of subjects (f/m)
PD 17 *or* 24 SS	Paired	Tone paired with 1.0 mA shock	Context Test	PD 17: 11 (5/6); PD 24: 9 (4/5)
PD 17 *or* 24 WS	Paired	Tone paired with 0.3 mA shock	Context Test	PD 17: 10 (5/5); PD 24: 10 (6/4)
PD 17 *or* 24 SS	Unpaired	Tone not paired with 1.0 mA shock	Context Test	PD 17: 12 (6/6); PD 24: 10 (4/6)
PD 17 *or* 24 WS	Unpaired	Tone not paired with 0.3 mA shock	Context Test	PD 17: 10 (6/4); PD 24: 9 (5/4)
PD 17 *or* 24 SS	Paired	Tone paired with 1.0 mA shock	Novel Context and Tone Test	PD 17: 8 (3/5); PD 24: 8 (4/4)
PD 17 *or* 24 WS	Paired	Tone paired with 0.3 mA shock	Novel Context and Tone Test	PD 17: 9 (5/4); PD 24: 9 (5/4)
PD 17 *or* 24 SS	Unpaired	Tone not paired with 1.0 mA shock	Novel Context and Tone Test	PD 17: 9 (6/3); PD 24: 8 (5/3)
PD 17 *or* 24 WS	Unpaired	Tone not paired with 0.3 mA shock	Novel Context and Tone Test	PD 17: 9 (4/5); PD 24: 8 (4/3)

Note: SS  =  Strong shock; WS  =  Weak shock

### Results and Discussion

#### Baseline Freezing

To use freezing as an index of fear requires observing an increase in the levels of freezing in the presence of a putative fear-inducing CS. This can be complicated when there are differences in baseline freezing levels. In these studies, initial freezing during habituation differed as a function of age F(1,73)  = 35.5, p<.05 (PD 17 = 17.7%, PD 24 = 2.0%) and shock level F(1,73)  = 4.11, p<.05 (weak shock  = 7.2%, strong  = 13.3%). The difference in baseline freezing by age was expected [42]. However, the difference in freezing by shock level must be due to chance, as rats were randomly assigned and at the time of habitation had received no stimulation. In contrast, freezing to the novel context occurred after the training session and served as a baseline for auditory freezing. In this case, there was a main effect of shock level F(1,59)  = 4.51, p<.05, with subjects receiving the stronger shock (M = 48.0%) freezing significantly more than subjects receiving the weaker shock (M = 37.6%). Although there were no effects of age, there was an age X condition interaction F(1,59)  = 4.91, p<.05, in which the paired subjects at PD 17 (M = 47.1%) showed greater freezing than the unpaired subjects (M = 39.0%), whereas at PD 24 the unpaired subjects (M = 49.4%) froze more than the paired subjects (M = 36.4%). There were no pairwise differences. As mentioned above, to assess auditory-cue induced freezing against these different baselines, difference scores were calculated and used for all further analyses (see methods).

#### Auditory Fear Conditioning

**Figure 4:**
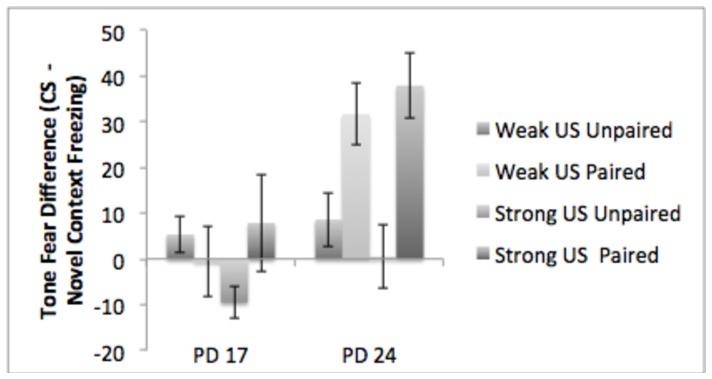
The results of Experiment 2 – auditory conditioning. Only PD 24 subjects exposed to paired CS-US presentations demonstrated subsequent enhanced freezing to the tone, regardless of shock intensity. PD 17 subjects and unpaired PD 24 subjects showed no effect of prior training.

We conducted a 2 (age: PD 17, PD 24) X 2 (condition: paired, unpaired) X 2 (shock intensity: 0.3 mA or 1.0 mA) ANOVA to look for effects and interactions of age, shock intensity, and condition on auditory conditioning (see Figure 4). Consistent with the hypothesis that PD 17 subjects demonstrate impaired conditioned freezing generally, this analysis revealed significant main effects for condition F(1, 59)  = 11.14, p<.01 and age F(1, 59)  = 14.53, p<.01, as well as a conditioning X age interaction F(1, 59)  = 5.17, p<.05. Post-hoc Tukey's tests revealed that the paired PD 24 subjects differed from all other groups, which did not differ from each other.

#### Contextual Fear Conditioning

**Figure 5:**
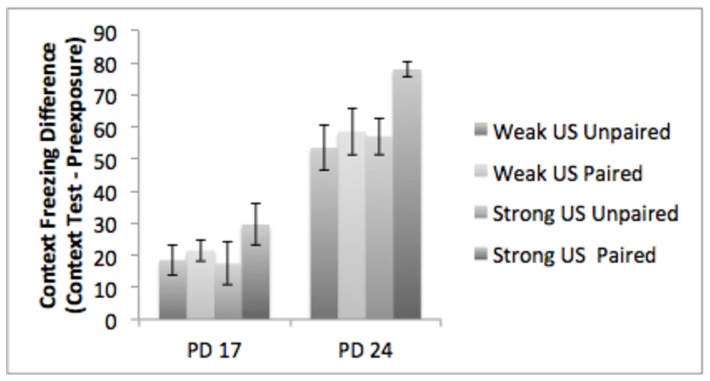
The results of Experiment 2 – contextual conditioning. PD 24 subjects showed significantly enhanced freezing to the conditioning context compared to PD 17 subjects regardless of shock intensity.

We also conducted a 2 (age: PD 17, PD 24) by 2 (condition: paired, unpaired) X 2 (shock intensity: 0.3 mA or 1.0 mA) ANOVA to look for effects and interactions of age, shock intensity, and condition on contextual conditioning (see Figure 5). Consistent with our earlier results, there was main effect of age F(1,73)  = 65.5, p<.01, demonstrating reduced freezing in the PD 17 subjects. There were no other significant effects or interactions.

## General Discussion

These experiments further investigate the ontogeny of fear conditioning in rats. Our data suggest that both auditory and contextual fear conditioning continue to emerge between PD 17 and 24, consistent with development occurring in the essential fear circuit. This is in contrast to some previous work suggesting that behavioral changes during this period were largely limited to contextual learning [7]–[10], but consistent with other work suggesting a more flexible timeline [21], [22], [25].

### Development of the Contextual Conditioning System

The current data suggest pre-exposure at PD 17 generally fails to enhance aversive contextual conditioning occurring at PD 18 regardless of the placement-to-shock interval, although there is evidence of context-induced freezing in these subjects at this age (Experiment 1–1, Experiment 2). This is consistent with previous work demonstrating that freezing to a conditioning context can be observed in pre-weaning rats, although conditioning tends to be weaker than in post-weanlings [7], [10], [14]. These data are in conflict with those recently reported by Pisano et al. (2012), in which pre-exposure to a context did enhance conditioning in PD 17 subjects. However, the enhancement they observed in the freezing response in their pre-weaning subjects was relatively small compared to their older subjects. In addition, we observe higher levels of freezing in our control subjects than they did, perhaps obscuring a similar increase.

Previous work has suggested that contextual learning around PD 17 can be more strongly elicited by enriching the context or conducting simultaneous auditory conditioning [21], [22], by neonatal handling [43], or prior exposure to footshock [24]. In addition, contextual fear in pre-weaning subjects appears more clearly when assessing fear-induced behavioral changes other than freezing [23] or by assessing fear immediately after conditioning [9]. Importantly, it has been suggested that there are at least two different cognitive strategies that subjects can use to acquire fear to a context: the configural learning system and the elemental learning system [36], [44]. The configural strategy involves the subject forming an integrated mental representation of the context, which can then be associated with the aversive stimulus and later induce the freezing response. The elemental strategy involves the subject forming an aversive associations with individual elements of the context, which can later induce freezing. These two types of learning produce the same behavioral changes, but only the configural strategy requires an intact hippocampus [36], [44]. Thus, we cannot assume that developing animals that appear to exhibit contextual fear conditioning are utilizing the “configural learning system” or the hippocampus in the absence of specific hippocampus manipulations. One way to avoid this problem is to use the contextual pre-exposure facilitation paradigm, which is highly sensitive to hippocampus damage (Foster & Burman, 2010; Rudy et al., 2002) and emerges with development (Schiffino et al. 2011). Therefore the presence of context-induced freezing implies some level of contextual fear conditioning is possible at PD 17, but the absence of a benefit from pre-exposure indicates that they this ability may not rely upon a “configural” learning strategy. The presence of context-induced freezing alone cannot be taken to imply a mature contextual learning system.

The findings at PD 17 stand in contrast to the effects of contextual exposure after weaning. Pre-exposure at PD 24 generally facilitated conditioning at PD 25, as shown by the significant effect of pre-exposure on all three dependent measures of fear (Experiment 1–2). Elevated levels of context-induced freezing were also observed following auditory fear conditioning at this age (Experiment 2). This is consistent with the large body of evidence that post-weaning rats demonstrate elevated levels of context-elicited freezing. However, we found little interaction between contextual pre-exposure and placement-to-shock interval. This is in contrast to work reported by Fanselow (1990), which demonstrated that pre-exposure only enhanced single-shock conditioning at intermediate delays (9 or 27 s) in two strains of adult rats. Conditioning with longer and shorter placement-to-shock intervals was unaffected. One explanation for Fanselow's findings is that at longer placement-to-shock intervals, the shock is no longer “immediate” and all subjects have time to learn about the context and reach asymptotic fear. At shorter placement-to-shock intervals, the benefits of the pre-exposure are also lost, perhaps because the subjects do not have time to recognize the context. Subsequent studies using adult [45]–[48] or developing [23], [38]–[40], [49]–[51] rats have largely chosen a single, short, placement-to-shock interval (usually 2–5 s), precluding similar analyses. In mice, pre-exposure appears to facilitate single-shock context conditioning with a variety of placement-to-shock intervals, including 2 s [52], 5 s [53], & 20 s [44], consistent with the current data, although it doesn't appear that this variable has been examined within a single experiment.

Finally, in contrast to the conclusions of Foster & Burman (2010), pre-exposure at PD 17 generally fails to facilitate contextual freezing following conditioning at PD 25. Foster & Burman (2010) made the observation that contextual exposure at PD 17 could facilitate fear conditioning at PD 24–25 even though it failed to do so at PD 18. This suggested that the developmental deficit at PD 17/18 was not in the ability to learn about a context, but rather in the ability to use contextual information for fear conditioning. Yap & Richardson (2005, 2007) make a similar point regarding latent inhibition and renewal of fear, suggesting that the ability to acquire, but not express, contextual learning exists prior to weaning. Although more recent evidence suggests that preweaning subjects do show contextual renewal in an odor-enhanced context [30] or when assessing taste aversion [29], whether these subjects are using a configural or an elemental strategy remains unclear. In an attempt to clarify this issue, the current data suggest that PD 17 subjects, unlike PD 24 subjects, generally show little benefit of contextual pre-exposure, regardless of when conditioning occurred. A specific examination of moderate placement-to-shock intervals did find an enhancement in freezing, consistent with our previous work (Foster & Burman, 2010), suggesting that we can replicate our previous findings when examining highly similar parameters. Nevertheless, our previous conclusion that contextual learning was similar at PD 17 and 24 appears to be incorrect. It is important to note that the current studies differ from Foster & Burman (2010) in the source of the rats (Harlan vs. Charles River), the colony and housing conditions, the fear conditioning equipment and the method of assessing freezing, all of which are likely important factors. Despite these differences, that we observe a similar enhancement of freezing when specifically examining similar placement-to-shock intervals suggests that our earlier findings were robust, but exist in a high limited parametric space involving a single dependent measure. Regardless, the conclusion that PD 17 and PD 23 subjects are equally able to form a contextual representation is clearly not supported by the current data.

Our confidence in the current results is increased because three measures of fear, all based on movement, were consistent in assessing the effects of pre-exposure on contextual fear conditioning. Duration of freezing behavior is perhaps the most common measure of fear conditioning in rodents and has been used for over 45 years [54]. For our studies, freezing behavior was assessed automatically using a load-cell system in which the lack of disruption below a certain threshold was defined as freezing. In addition, the latency to begin freezing was also recorded. Our assumption is that the latency to begin freezing reflects the ability of the subject to recognize the context as dangerous and furthermore that this is representative of the degree of their familiarity with the environment. This measure has been previously used to measure fear conditioning with latencies similar to those reported here [55], [56] and in studies assessing other types of fear [57]. Finally, average cage displacement prior to the first freezing bout is a measure of the amplitude of movement. Although this specific metric has not been reported as a measure of fear to the best of our knowledge, that fear can suppress locomotion is well established [58]. The current data confirm fear can suppress movement even in the absence of freezing.

Overall, the current data clearly demonstrate that context-induced freezing is impaired in rats at PD 17 compared to rats at PD 24.

### Development of the Fear Conditioning System

Although the current studies were initially designed to examine the development of contextual fear conditioning, Experiment 2 also demonstrated a relative impairment in auditory fear conditioning at PD 17 relative to PD 24. Although this initially appears to be a novel finding, in conflict with previous literature [7]–[10], [59], it has been reported indirectly in several previous studies [25], [32], [33]. Even these findings are in contrast to the complete lack of conditioning in PD 17 subjects in our hands. One difference is that our study uses a continuous 4 kHz pure tone, a stimulus clearly capable of activating the auditory pathway in PD 17 rats [60], [61]. Other studies showing differences in auditory conditioning at these ages also use continuous sounds [25], [32], [33], although not the same frequency used here. Studies that demonstrated similar levels of conditioning in pre- and postweaning subjects often used pulsing or intermittent clicks or tones [7], [8], [59], a manipulation known to affect the neural circuitry required for fear conditioning [62] and that may affect stimulus salience. Other husbandry differences may also play a role. Early stress [24], pain [63], enrichment [64], and bedding [65] all play a key role in the emergence of fear. Therefore, seemingly inconsequential aspects of husbandry (tattooing for identification; background noise or activity in colony; in-house colonies vs. timed pregnant ordering) may turn out to be important influences in the emergence of this task. Nevertheless, the parameters used here are clearly capable of producing robust conditioning, just not in PD 17 subjects, suggesting the development of fear conditioning circuitry is still occurring at this age.

The current findings suggest that the development of contextual learning during this period may be secondary to development occurring in the fear conditioning circuit more generally. Although the current data cannot rule out development of sensory or motor systems as contributing to the behaviors we observe, the increase in conditioned freezing between PD 17 and 24 is consistent with the known development of the relevant limbic circuitry. Indeed, a variety of amygdalar nuclei continue to develop well past this period in terms of volume, cell number and especially the number of oligodendrocytes [66]. In addition, the electrophysiological properties of basolateral amygdala neurons continue to change until at least PD 28 [67]. Given the critical role of the amygdala in fear conditioning [68], [69], this development is consistent with the behavioral changes we see. However, development in other regions would also be consistent with these behavioral changes. For example, there is an emerging understanding that different hippocampus subregions may have different roles in fear conditioning. Although the dorsal hippocampus may specialize in spatial/contextual learning (but see [70]), and thus have a specific role in contextual learning, lesion studies that include the ventral hippocampus also indicate that this region may be involved in fear and anxiety more generally [71]–[77]. Indeed, a periweaning developmental period for fear is consistent with the emergence of LTP in the dentate gyrus [78], [79]. Thus, depending on whether hippocampus development is uniform across the septo-temporal axis, an immature hippocampus would be expected to produce impairments in both contextual and auditory fear conditioning.

Even though there are clearly situations in which rats at the 3^rd^ and 4^th^ weeks of life can be shown to be equivalent, there is now sufficient evidence that there exist other situations in which fear conditioning differs at these ages. Once differences in fear conditioning as a function of age have been shown, that other parameters exist in which conditioning is equivalent does not affect our overall conclusion that age (perhaps interacting with other variables) determines the emergence of fear conditioning during this developmental period. The current data support the overall conclusion that critical development is occurring in the fear conditioning circuit between PD 17 and 24.

## Supporting Information

Checklist S1 NC3R Guidelines Checklist.
(PDF)
